# Minimally Invasive Surgery Versus Conventional Craniotomy for Spontaneous Hypertensive Supratentorial Hemorrhage: A Systematic Review and Meta-Analysis of Randomized Controlled Trials

**DOI:** 10.7759/cureus.112833

**Published:** 2026-07-17

**Authors:** Muhammad Irfan, Muhammad Usman Shaikh, Muhammad Ahsan Shaikh, Syeda Rashid, Behram K Ghazi, Farwa Amjad, Muhammad Sajjad, Rizwan Haider

**Affiliations:** 1 Neurosurgery, Nishtar Medical University, Multan, PAK; 2 Internal Medicine, Dow University of Health Sciences, Karachi, PAK; 3 Medicine and Surgery, Chandka Medical College, Shaheed Mohtarma Benazir Bhutto Medical University (SMBBMU), Larkana, PAK; 4 Medicine and Surgery, Mohtarma Benazir Bhutto Shaheed Medical College, Karachi, PAK; 5 Medicine and Surgery, Faisalabad Medical University, Faisalabad, PAK; 6 Pediatric Medicine, Faisalabad Medical University, Faisalabad, PAK; 7 Internal Medicine, Combined Military Hospital (CMH) Lahore Medical College and Institute of Dentistry, Lahore, PAK

**Keywords:** functional outcome, meta-analysis, minimally invasive surgery, mortality, open craniotomy, spontaneous intracerebral hemorrhage

## Abstract

The optimal surgical approach for spontaneous hypertensive intracerebral hemorrhage (ICH) remains controversial. Conventional craniotomy has failed to demonstrate consistent benefit, whereas minimally invasive surgery (MIS) has emerged as a promising alternative. Whether outcomes differ according to MIS technique remains unclear. This study aimed to compare functional and mortality outcomes of MIS vs. conventional craniotomy for spontaneous hypertensive ICH and to evaluate technique-specific effects through subgroup and interaction analyses. A systematic review and meta-analysis of randomized controlled trials was conducted in accordance with the Preferred Reporting Items for Systematic Reviews and Meta-Analyses 2020 guidelines. PubMed, Embase, and Cochrane Central Register of Controlled Trials were searched from inception to February 2026. Adult patients with spontaneous hypertensive supratentorial ICH undergoing MIS or conventional craniotomy were included. The primary outcome was a favorable functional outcome (modified Rankin Scale ≤2 or ≤3). Secondary outcomes included mortality. Risk ratios (RRs) with 95% confidence intervals (CIs) were pooled using a random-effects model. Prespecified subgroup analyses were performed by MIS technique (endoscopic evacuation vs. stereotactic aspiration), and interaction testing was conducted. Three randomized trials encompassing 925 patients (592 in the MIS group and 333 in the craniotomy group) were included. Pooled analysis demonstrated no significant difference in mortality between MIS and craniotomy (RR, 0.84; 95% CI, 0.60-1.19; p = 0.33; I² = 0%). However, MIS was associated with a significantly higher likelihood of favorable functional outcome compared with craniotomy (RR, 1.52; 95% CI, 1.23-1.87; p < 0.0001; I² = 0%). Subgroup analysis revealed that endoscopic techniques significantly improved functional outcomes (RR, 1.52; 95% CI, 1.20-1.93; p = 0.0006), while stereotactic aspiration demonstrated a favorable but less pronounced effect. MIS improves functional outcomes compared with conventional craniotomy in spontaneous hypertensive ICH. Endoscopic techniques provide greater functional benefit than stereotactic aspiration, while mortality remains unchanged. These findings support a technique-specific approach to surgical management of ICH.

## Introduction and background

Spontaneous hypertensive intracerebral hemorrhage (ICH) remains one of the most devastating subtypes of stroke, accounting for approximately 10%-15% of all cerebrovascular events worldwide and carrying disproportionately high rates of mortality and long-term disability [1,2]. Despite advances in neurocritical care and medical management, outcomes remain poor, with up to 40% of patients dying within the first month and a substantial proportion of survivors remaining functionally dependent [3].

Conservative medical management remains the mainstay of treatment for spontaneous hypertensive ICH. Surgery is reserved for selected patients with neurological deterioration, significant mass effect, elevated intracranial pressure, or cerebellar hemorrhage with brainstem compression or hydrocephalus. These limitations of medical therapy have driven the development of minimally invasive surgical techniques to improve outcomes while reducing surgical trauma. Surgical evacuation of an intracerebral hematoma has long been considered a potential strategy to reduce mass effect, limit secondary brain injury, and improve neurological recovery. Conventional craniotomy has historically been the standard surgical approach; however, major randomized controlled trials (RCTs), including the Surgical Trial in Intracerebral Hemorrhage (STICH) and STICH II, failed to demonstrate a clear functional or survival benefit of early open surgery compared with conservative management [4,5]. These neutral results have been attributed in part to the invasiveness of craniotomy, which may exacerbate perihematomal injury through extensive cortical disruption, brain retraction, and surgical trauma [6]. Therefore, minimally invasive surgical (MIS) techniques have been developed to achieve effective hematoma evacuation with minimal collateral brain injury, and these approaches include endoscopic hematoma evacuation, stereotactic aspiration with or without thrombolysis, and, more recently, minimally invasive parafascicular surgery [7]. Experimental and clinical studies suggest that reducing surgical invasiveness may limit secondary neuronal injury, decrease perihematomal edema, and promote improved functional recovery [8].

Several RCTs have evaluated minimally invasive surgery (MIS) for spontaneous hypertensive ICH using functional outcome measures such as the modified Rankin Scale (mRS) and Glasgow Outcome Scale. Multiple RCTs were conducted to evaluate the MIS strategy for ICH, such as the Minimally Invasive Surgery with Thrombolysis in Intracerebral Hemorrhage Evacuation III trial, which showed that while the primary endpoint was not met, greater hematoma volume reduction was strongly associated with improved functional outcomes (mRS 0-3 at one year), underscoring the importance of effective clot evacuation [9]. More recently, the Early Minimally Invasive Removal of Intracerebral Hemorrhage (ENRICH) trial reported significantly improved utility-weighted functional outcomes with early minimally invasive parafascicular surgery compared with medical management alone [10]. However, ENRICH did not include a direct comparison with conventional craniotomy. Despite increasing interest in MIS, existing evidence remains heterogeneous, and many prior meta-analyses have grouped all MIS techniques as a single intervention. This approach overlooks important technical and biological differences between endoscopic evacuation and stereotactic aspiration, which may result in differential effects on functional recovery and survival. Furthermore, mortality outcomes have been inconsistently reported across trials, and the relative efficacy of MIS vs. craniotomy remains uncertain. Therefore, we conducted a systematic review and meta-analysis of RCTs to directly compare MIS with conventional craniotomy for spontaneous hypertensive ICH. We aimed to assess functional and mortality outcomes and to perform prespecified subgroup and interaction analyses to determine whether treatment effects vary according to MIS technique. By focusing on technique-specific comparisons, this study seeks to provide clinically relevant evidence to inform surgical decision-making in patients with spontaneous hypertensive ICH.

## Review

Methods

This systematic review and meta-analysis was conducted in accordance with the Preferred Reporting Items for Systematic Reviews and Meta-Analyses (PRISMA) 2020 guidelines [11]. A protocol was developed to define eligibility criteria, outcomes, and analytical methods. Formal registration was not performed.

Eligibility Criteria

Study design: We included RCTs comparing minimally invasive surgical techniques with conventional craniotomy for spontaneous ICH.

Participants: Only trials enrolling patients with primary spontaneous hypertensive supratentorial ICH were included. During full-text screening, we confirmed that each eligible study excluded secondary causes of ICH, including traumatic hemorrhage, aneurysms, arteriovenous malformations, brain tumors, hemorrhagic transformation of ischemic infarction, and other structural or vascular causes, according to each study's predefined eligibility criteria.

Interventions and comparators: Eligible interventions included minimally invasive surgical techniques such as endoscopic hematoma evacuation, stereotactic aspiration, and minimally invasive parafascicular surgery, with comparators including conventional craniotomy.

Outcomes

The primary outcome was a favorable functional outcome, defined as an mRS of ≤2 or ≤3, at the longest reported follow-up (typically six months). Secondary outcomes included all-cause mortality at the longest reported time point and procedure-related complications.

Search Strategy

A systematic literature search was conducted in PubMed/MEDLINE, Embase, and the Cochrane Central Register of Controlled Trials from database inception to February 2026. Database-specific search strategies combined controlled vocabulary (Medical Subject Headings for PubMed and Emtree for Embase) with free-text terms related to spontaneous ICH, hypertensive ICH, MIS, endoscopic evacuation, stereotactic aspiration, and craniotomy using Boolean operators (AND/OR). Reference lists of eligible studies and relevant systematic reviews were manually screened to identify additional studies.

Study Selection

Two reviewers independently screened titles and abstracts for eligibility. Full texts of potentially relevant studies were reviewed independently to determine final inclusion. Discrepancies were resolved by discussion and consensus.

Data Extraction

Two reviewers independently extracted data using a standardized data extraction form. Extracted variables included the following: study characteristics (author, year, country, design), patient demographics and sample size, surgical technique, comparator functional outcome, mortality data, and follow-up duration. For multiarm trials, intervention groups were treated as independent comparisons by splitting the shared control group to avoid double-counting.

Risk of Bias Assessment

Risk of bias was assessed independently by two reviewers using the Cochrane Risk of Bias 2 (RoB 2) tool, evaluating the following: bias arising from the randomization process, bias due to deviations from intended interventions, bias due to missing outcome data, bias in outcome measurement, and bias in selection of the reported result. Each domain was rated as low risk, some concerns, or high risk. Disagreements were resolved by consensus.

Data Synthesis and Statistical Analysis

Meta-analyses were performed using Review Manager, version 5.4 (The Cochrane Collaboration, London, UK). Dichotomous outcomes were pooled using risk ratios (RRs) with 95% confidence intervals (CIs) under a random-effects model. Statistical heterogeneity was assessed using the I² statistic, with values >50% indicating substantial heterogeneity. Subgroup analyses were conducted based on the type of minimally invasive surgical technique: endoscopic hematoma evacuation and stereotactic aspiration. A test for subgroup differences (interaction) was performed to assess whether treatment effects differed between MIS techniques.

Results

Study Selection

**Figure 1:**
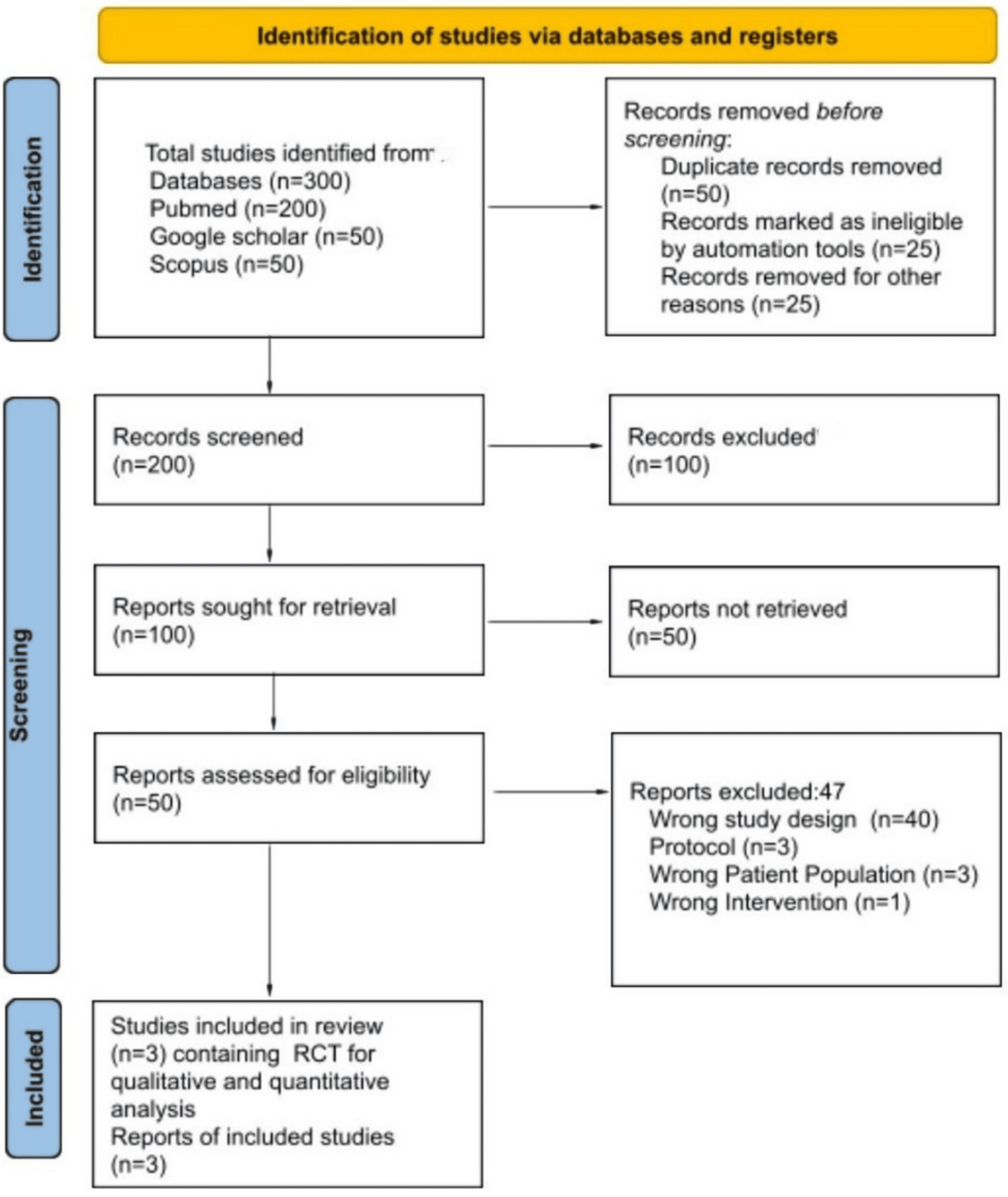
PRISMA 2020 flow diagram showing the search of databases and registers for the current meta-analysis PRISMA: Preferred Reporting Items for Systematic Reviews and Meta-Analyses; RCT: randomized controlled trials

During the search strategy, 300 articles were selected from the database, and screening was performed based on titles and abstracts. Out of the 300 articles, 50 duplicates were removed, in addition to 25 marked as ineligible by automation tools and 25 removed for other reasons. With 200 articles left, 100 records that did not meet the eligibility criteria were excluded; hence, 100 articles were sought for retrieval, of which 50 were not retrieved, leaving only 50 articles assessed for eligibility. Of the 50 articles, 47 records were excluded: 40 due to the wrong study design, three due to the wrong protocol, three due to the wrong patient population, and one due to the wrong intervention, as shown in the PRISMA flow diagram (Figure 1). The studies by Xu et al. [12], Feng et al. [13], and Cho et al. [14] were included for quantitative and qualitative analyses.

Risk of Bias and Certainty of Evidence

**Figure 2:**
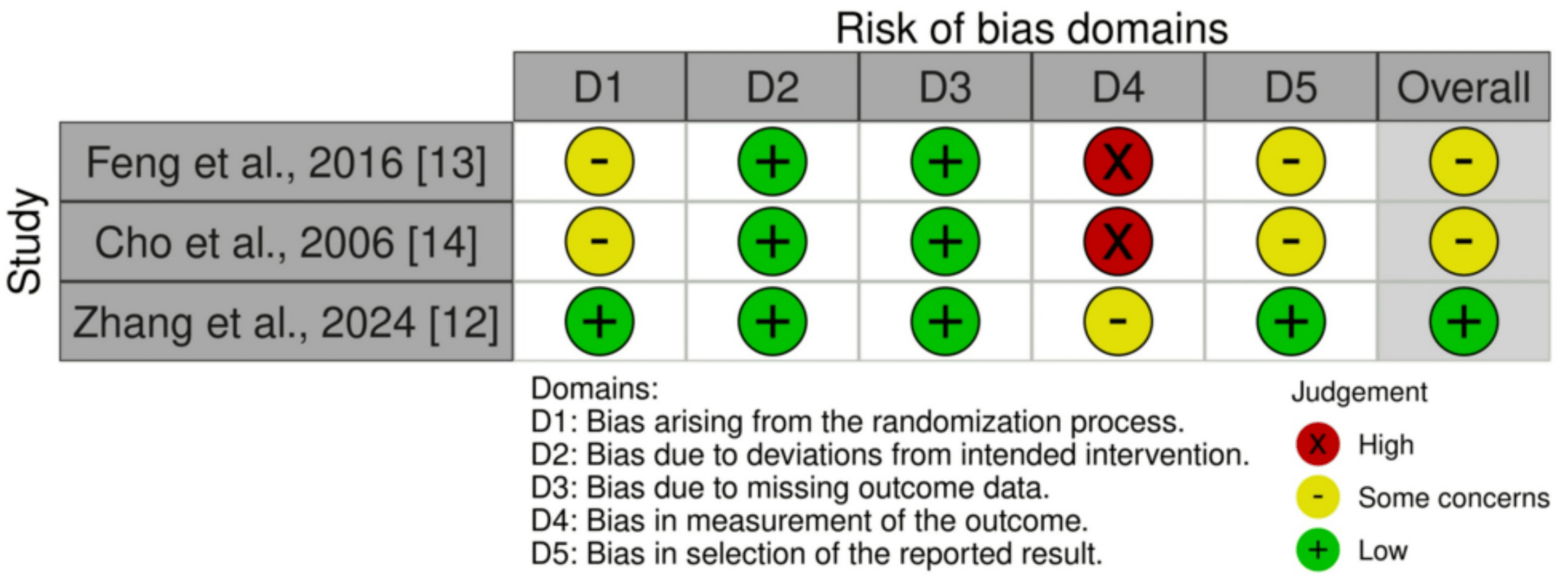
RoB2 tool for the randomized controlled trials included in the meta-analysis RoB2: risk of bias 2

**Figure 3:**
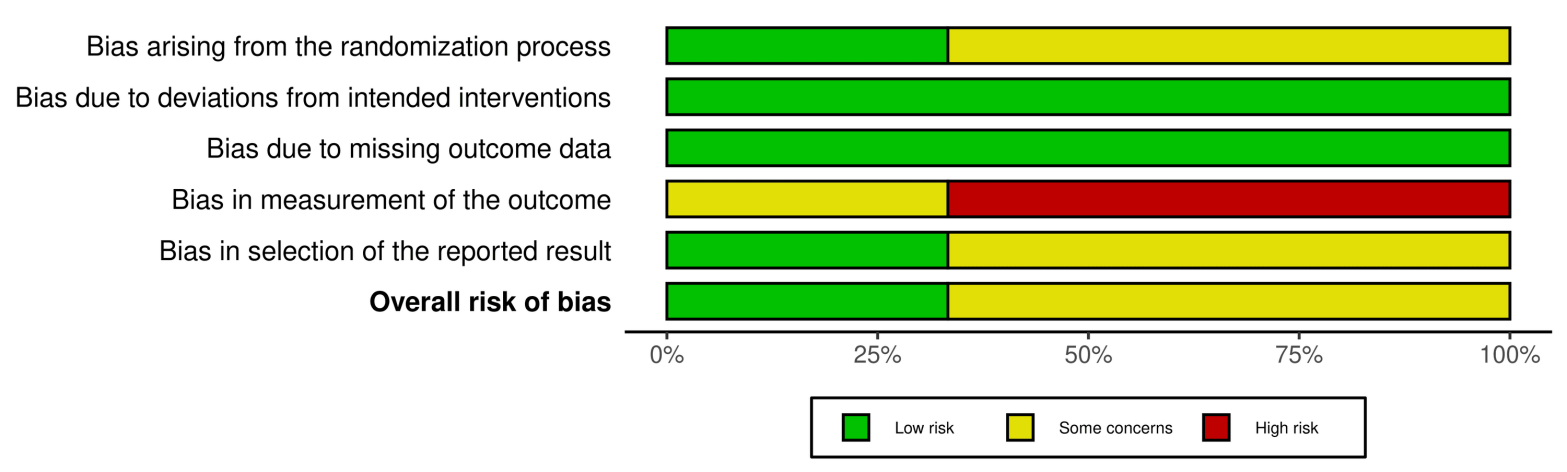
RoB2 risk of bias assessment summary plot for the randomized controlled trials included in the meta-analysis RoB2: risk of bias 2

The RoB2 tool indicated that Xu et al. had a low overall risk of bias [12]. However, Feng et al. [13] and Cho et al. [14] had some overall concerns because of biases arising from the randomization process, measurement of the outcome, and selection of reported results (Figures 2, 3).

Characteristics of Included Studies

A total of three RCTs comprising 925 patients were included in the analysis. Of these, 592 patients underwent minimally invasive surgical procedures, including endoscopic evacuation and stereotactic aspiration, while 333 patients underwent conventional craniotomy. The mean age of participants ranged from approximately 55 to 69 years across studies. Most hemorrhages were located in supratentorial or basal ganglia regions. Functional outcomes were assessed using various scales, including the mRS, Activities of Daily Living (ADL) [15], and Functional Independence Measure (FIM) [16], with a follow-up duration of approximately 6 months across all studies (Table 1).

**Table 1:** Characteristics of included trials MIS: minimally invasive surgery; RCT: randomized controlled trial; ICH: intracerebral hemorrhage; mRS: modified Rankin Scale; ADL: Activities of Daily Living; FIM: Functional Independence Measure

Study	Year	Country	Study design	Sample size (MIS/craniotomy)	Mean age (years)	Hematoma location	Intervention (MIS type)	Comparator	Outcome measures	Follow-up
Xu et al. [12]	2024	China	Multicenter RCT	439/212	~58-60	Supratentorial	Endoscopic + stereotactic aspiration	Craniotomy	mRS, mortality	6 months
Feng et al. [13]	2016	China	RCT	93/91	~66-69	Hypertensive ICH	Endoscope-assisted keyhole	Craniotomy	ADL, mortality	6 months
Cho et al. [14]	2006	Taiwan	RCT	60/30	~55-56	Basal ganglia	Endoscopic + stereotactic aspiration	Craniotomy	FIM, Barthel, mortality	6 months

Outcomes

Mortality outcomes were reported in three included trials. Trials comparing MIS with craniotomy reported six-month mortality and another outcome, which was a functional outcome. Two trials are split into two separate arms on the basis of endoscopic and stereotactic aspiration.

Overall Analysis (MIS vs. Craniotomy)

**Figure 4:**
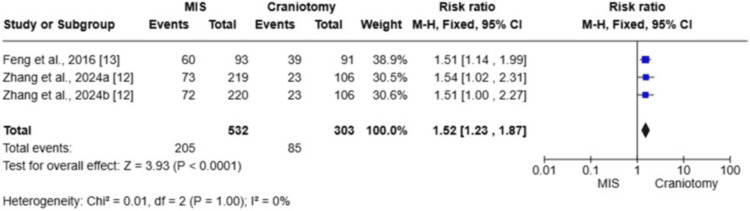
Forest plot demonstrating improved favorable functional outcomes with MIS compared to craniotomy MIS: minimally invasive surgery; CI: confidence interval; M-H: Mantel-Haenszel

Three RCTs involving 925 patients were included in the quantitative synthesis. Pooled analysis of functional outcomes demonstrated that MIS was associated with a significantly higher likelihood of a favorable functional outcome compared with conventional craniotomy (RR, 1.52; 95% CI, 1.23-1.87; p < 0.0001). There was no observed heterogeneity among studies (I² = 0%), indicating consistent effects across trials (Figure 4).

Secondary Outcome: Mortality

**Figure 5:**
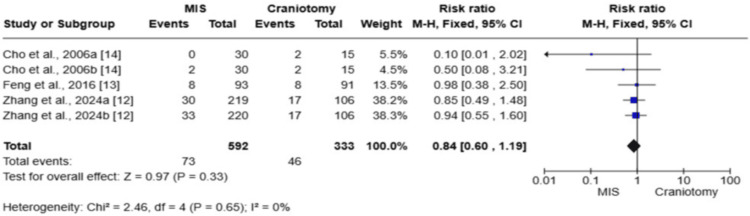
Forest plot showing mortality outcome for MIS vs. craniotomy MIS: minimally invasive surgery; CI: confidence interval; M-H: Mantel-Haenszel

Overall analysis (MIS vs. craniotomy): Pooled analysis of RCTs demonstrated no significant difference in mortality between MIS and conventional craniotomy (RR, 0.84; 95% CI, 0.60-1.19; p = 0.33). There was no observed heterogeneity among studies (I² = 0%), indicating consistent findings across trials (Figure 5).

Subgroup Analysis by MIS Technique

**Figure 6:**
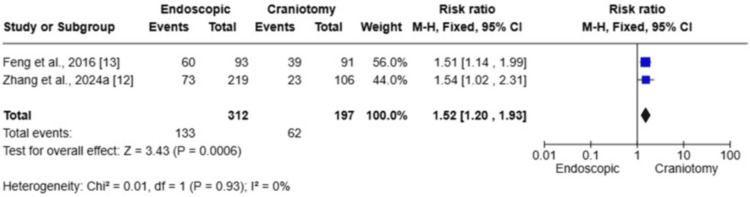
Forest plot for subgroup analysis of endoscopic MIS vs. craniotomy showing primary functional outcome MIS: minimally invasive surgery; CI: confidence interval; M-H: Mantel-Haenszel

Endoscopic MIS vs. craniotomy: In the endoscopic subgroup analysis, endoscopic MIS was associated with a significantly higher likelihood of a favorable functional outcome compared to craniotomy (RR, 1.52; 95% CI, 1.20-1.93; p = 0.0006), with no observed heterogeneity (I² = 0%) (Figure 6).

Stereotactic Aspiration MIS vs. Craniotomy

**Figure 7:**
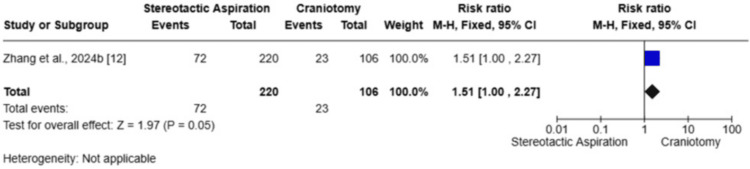
Forest plot showing subgroup analysis of stereotactic aspiration vs. craniotomy for functional outcome CI: confidence interval; M-H: Mantel-Haenszel

Based on the single available randomized study [12], stereotactic aspiration was associated with a higher likelihood of favorable functional outcome compared with craniotomy (RR, 1.51; 95% CI, 1.00-2.27; p = 0.05). As only one study contributed to this comparison, heterogeneity could not be assessed (Figure 7).

Publication Bias

Formal assessment of publication bias using funnel plots was not performed due to the small number of included trials.

Discussion

This systematic review and meta-analysis of RCTs demonstrates that MIS significantly improves functional outcomes compared with conventional craniotomy in patients with spontaneous hypertensive ICH; however, subgroup analysis indicates that this benefit is dependent on techniques such as endoscopic evacuation, which provides greater functional improvement than stereotactic aspiration, whereas there was no significant reduction in mortality.

Functional Outcomes and Mechanistic Insights

The observed improvement in functional outcomes with MIS highlights the importance of minimizing secondary brain injury during hematoma evacuation, and conventional craniotomy is associated with substantial cortical disruption, worsening perihematomal injury, and neurological deficits. Alternatively, in MIS techniques, smaller surgical corridors were used, resulting in minimal tissue manipulation and thereby preserving surrounding brain structures [17,18]. The superiority of endoscopic MIS over stereotactic aspiration in our subgroup analysis is consistent with prior literature demonstrating that direct visualization enables more complete hematoma evacuation and improved hemostasis [19]. Studies have shown that achieving a lower residual hematoma volume is strongly correlated with improved functional recovery, supporting the mechanistic basis for our findings [20].

Comparison With Existing Evidence

Our findings are consistent with previous systematic reviews and observational studies, suggesting a benefit of minimally invasive approaches over conventional surgery.

Although a recent meta-analysis comprehensively evaluated MIS for spontaneous ICH, uncertainty remains regarding its comparative effectiveness vs. conventional craniotomy and whether outcomes differ according to specific minimally invasive techniques, providing the rationale for the present focused analysis [21].

A meta-analysis by Huan et al. reported that less invasive surgical techniques were associated with improved outcomes compared with craniotomy, although heterogeneity in included studies limited definitive conclusions [22]. Similarly, it has been demonstrated that endoscopic evacuation may result in better functional outcomes and reduced complications compared with open surgery, as a meta-analysis involving 1,652 patients revealed that intraoperative blood loss, hospitalization days, and complications were significantly lower than in the conventional craniotomy group [23]. Recent cohort studies have also reinforced the potential advantages of MIS, as they reported favorable outcomes with minimal endoscopic surgery, emphasizing reduced operative trauma and improved clot evacuation [24].

These findings correlate with our results, demonstrating a clear functional benefit of MIS, particularly with endoscopic techniques. Importantly, many prior analyses have grouped MIS techniques together. Our study adds to the literature by showing that not all MIS approaches yield equivalent outcomes, highlighting the importance of surgical technique selection by performing a subgroup and interaction analysis.

Mortality Outcomes

Although MIS had a great impact on improvement in functional outcome, it did not significantly reduce mortality compared with craniotomy. This finding has been elaborated on in prior studies, which show that mortality is mainly driven by baseline factors such as hematoma size, location, and neurological status at presentation [25]. Therefore, surgical intervention may have a greater impact on functional recovery among survivors than on overall survival.

Clinical Implications

These findings support a shift toward minimally invasive surgical approaches, particularly endoscopic evacuation, in the management of spontaneous hypertensive ICH. In clinical practice, the choice of surgical technique should consider not only feasibility and resource availability but also the potential for maximizing functional recovery. Furthermore, our results highlight the need for standardization of MIS techniques and training, as variability in surgical expertise may influence outcomes.

Limitations

This analysis has several limitations. The number of included randomized trials was limited, and subgroup analyses were based on a small number of studies. Differences in outcome definitions, surgical timing, perioperative care, and functional outcome measures (mRS, ADL, and FIM/Barthel) may have introduced variability. Additionally, the inability to blind surgical interventions introduces potential bias. Finally, long-term cognitive outcomes and quality-of-life measures were not consistently reported.

Future Directions

For future research, there should be direct comparisons of different MIS techniques, particularly endoscopic evacuation vs. stereotactic aspiration, using standardized outcome measures. In addition, imaging biomarkers, surgical optimal timing evaluation, and cost-effectiveness analysis should be considered for further refinement of patient selection and clinical decision-making.

## Conclusions

In contrast to earlier trials of open surgery, contemporary randomized evidence indicates that MIS, particularly endoscopic evacuation, significantly improves functional outcomes compared with conventional craniotomy in spontaneous hypertensive ICH. These findings highlight the importance of surgical technique selection and support the preferential use of endoscopic MIS in appropriate patients.
